# Consumer Acceptability of Various Gluten-Free Scones with Rice, Buckwheat, Black Rice, Brown Rice, and Oat Flours

**DOI:** 10.3390/foods14142464

**Published:** 2025-07-14

**Authors:** Jihyuk Chae, Sukyung Kim, Jeok Yeon, Sohui Shin, Seyoung Ju

**Affiliations:** Department of Food & Nutrition, College of Biomedical and Health Science, Konkuk University, Chungju 27478, Republic of Korea

**Keywords:** gluten-free scone, consumer acceptability, check-all-that-apply, liking, partial least squares regression (PLSR) analysis

## Abstract

Due to consumer needs and the prevalence of gluten-related disorders such as celiac disease, the gluten-free food market is expanding rapidly and is expected to surpass USD 2.4 billion by 2036. The objective of this study was to substitute wheat flour with oat, black rice, brown rice, buckwheat, and rice flours in the production of gluten-free scones, to assess consumer acceptability, and to identify factors contributing to consumer acceptability using check-all-that-apply questions. The 10 attributes of appearance, color, texture, grainy flavor, sweetness, familiar flavor, novelty, familiarity, moistness, and consistency exhibited statistically significant differences among the samples (*p* < 0.001). One hundred consumers evaluated 18 attributes using a nine-point hedonic scale, and all attributes demonstrated statistically significant differences across six samples (*p* < 0.001). The samples from buckwheat and wheat scored the highest in consumer acceptability. The results indicate a strong positive correlation between overall liking and purchase intention, with sensory attributes such as nutty flavor, cohesiveness, appearance, moistness, color, texture, and inner softness positively influencing consumer acceptability. The attributes affecting negatively were thick throat sensation, unique flavor, and stuffiness. This study is expected to provide data to aid in the development of better-tasting gluten-free products that meet customer and market needs.

## 1. Introduction

Gluten is a structural protein formed by combining gliadin and glutenin in certain grains such as wheat, barley, and rye [1]. It plays a crucial role in creating desirable textures and volumes in baked products by holding carbon dioxide and binding other ingredients [2,3]. Wheat flour, which predominantly contains gluten, is primarily used as a key ingredient in everyday foods due to these critical functionalities.

However, celiac disease, an immune-related systemic disorder, is known to cause inflammation in the small intestine’s mucosal cells due to gluten, damaging the villi and resulting in malabsorption and atopy [1]. The demand for and production of gluten-free products are on the rise, not only because of celiac disease but also due to conditions like gluten ataxia, dermatitis herpetiformis, and wheat-related allergies [4]. The gluten-free food market is projected to expand at an annual rate of 9.8%, surpassing USD 800 million in 2023 and expected to exceed USD 2.4 billion by 2036 [5]. Nonetheless, gluten-free products remain less accessible to consumers due to their rarity and high costs. The ingredients commonly used in gluten-free products, such as oats, buckwheat, quinoa, and brown rice, typically contain higher levels of fiber and minerals than those in wheat-based products. Buckwheat, highly researched for its use in gluten-free products, contains phytochemicals named rutin and quercetin, which may have functional activities. Currently, it is very difficult to replace natural proteins that have similar rheological properties to those of gluten because gluten plays a crucial role in the essential viscoelastic properties for the texture and taste of bakery products. Therefore, acceptable and affordable gluten-free bakery products are scarce due to challenges in achieving the appropriate texture and volume [3]. To address these issues, ongoing research aims to enhance the quality and development of gluten-free bakery products like cookies, crackers, biscuits, and breads [6,7,8,9,10,11,12,13,14].

Originating in England, a scone is a type of quick bread made with butter, milk, egg, and sugar, leavened swiftly using baking powder without a fermentation process [10,15]. Due to this rapid preparation process and convenience, the consumption of scones as a meal substitute is steadily increasing in Republic of Korea, with various ingredients incorporated to enhance the flavor and health benefits [8]. Consequently, numerous studies have aimed to substitute wheat flour, the primary ingredient in scones, with gluten-free alternatives such as rice flour, oatmeal, and almond powder. Additionally, research to enhance functionality or taste by incorporating other ingredients has also been explored [10,15,16,17,18,19].

Check-all-that-apply (CATA) questions are a popular sensory evaluation technique that rapidly and easily collects sensory information from consumers, introduced in 2007 to gauge consumer perceptions of products [20,21,22,23]. CATA questions comprise a list of sensory terms and attributes from which panelists select all that they consider applicable to each sample [24,25]. Although the CATA technique involves simple and limitless multiple-choice questions for identifying relevant sensory attributes, the results are highly reliable for understanding the consumer acceptability of products and their profiles, similarly to those obtained from descriptive analyses conducted by trained panelists [26]. Previous studies on gluten-free bakery products have evaluated sensory attributes using a small panel of 7~30 participants [6,8,9,10,11]. Di Cairano et al. [20] assessed both experimental and commercial gluten-free biscuits for acceptability and sensory profiles using CATA questions.

In this study, we used gluten-free flours such as oat, black rice, brown rice, buckwheat, and rice flours for making gluten-free scones and identified factors contributing to consumer acceptability using CATA questions with 100 consumers. The purpose of this study is to assess whether flours from gluten-free flours, usable as gluten-free ingredients, evaluate their potential based on the product’s sensory qualities. Our research could serve as foundational information for developing more palatable and consumer-acceptable gluten-free bakery products and is expected to aid in ongoing future research.

## 2. Materials and Methods

### 2.1. Materials

The experiment used the following materials: wheat flour (CJ Jeiljedang, Yangsan, Gyeongsangnam-do, Republic of Korea), oat flour (Canadian oats milling, Duagh, AB, Canada), black rice flour (Green natural, Jindo, Jeollanam-do, Republic of Korea), brown rice flour (Color food, Anyang, Gyeonggi-do, Republic of Korea), buckwheat flour (Bongpyeongmemil, Pyeongchang, Gangwon-do, Republic of Korea), rice flour (Daedu, Gunsan, Jeollabuk-do, Republic of Korea), non-salted butter (Anchor, Sturgeon, Auckland, New Zealand), white sugar (Samyang, Ulsan, Republic of Korea), milk (Seoul Milk, Seoul, Republic of Korea), fine sea salt (CJ Jeiljedang, Sinan, Jeollanam-do, Republic of Korea), baking powder (Pungjeon, Incheon, Republic of Korea), and egg (Mokdong Farm, Yeongju, Gyeongsangbuk-do, Republic of Korea). The five types of flour selected for the gluten-free scones experiment were based on prior studies and are commonly used in Republic of Korean bakery products [10,13,16,17,18,19]. These 12 materials were purchased online “https://www.coupang.com (accessed on 12 September 2024)”.

### 2.2. Preparation of Samples

The experimental design of this study was informed by prior gluten-free bakery research [10,16,17,18,19,20]. Scone baking trials were carried out in a laboratory, and processes such as dough mixing, processing, and baking were conducted using laboratory equipment. Six types of scones were prepared: one control sample using original wheat flour, and five gluten-free variants using different flours (oat, black rice, brown rice, buckwheat, and rice flours). The mixing ratio and method were based on previous studies and preliminary experiments [10,16,17,18,19]. Preliminary tests used a recipe from prior related research to prepare wheat-flour scones, which were then evaluated. The composition and mixing ratio for the scones in this study were determined from several preliminary tests and are detailed in Table 1. The dry ingredients (flour, sugar, baking powder, and salt) were combined and sieved. Butter pieces were incorporated into the dry mixture until the mixture resembled large, coarse crumbs the size of small peas, using a food processor. Milk and eggs were added to moisten the dry ingredients. The dough was kneaded gently a few times until cohesive, then wrapped and left to rest in the refrigerator for 1 h. After resting, the dough was rolled out and the scones were cut into a square (4 cm × 5 cm), approximately 2 cm thick, placed on a baking pan, and baked in an oven (iCombi Pro, Rational, Berlin, Germany) at 180 °C for 15 min. The scones were then cooled, wrapped in plastic bags, and presented to consumers for sensory testing.

### 2.3. Consumer Test: Consumer Acceptability and CATA

The consumer testing of scones involved 100 consumers (females: 65, males: 35, age: 20~60 years) from the Chungju area (Chungcheongbuk-do, Republic of Korea). Participants were recruited using school bulletin boards, bulletin boards of public institutions, and websites of community. In terms of age, 30 participants were in their 20s, 20 in their 30s, 28 in their 40s, and 22 in their 50s. Participants were not required to have prior experience in sensory testing, but they should not have had any aversions to eating scones or allergies to any of the ingredients. This study received approval from the Institutional Review Board of Konkuk University (7001355-202405-HR-794).

All samples were served in a white paper dish (10 cm in diameter) with strawberry jam (Ottogi, Anyang, Gyeonggi-do, Republic of Korea) accompanied by drinking water to cleanse their mouths between samples and minimize residual effects. These samples, coded with random three-digit numbers, were issued using a Latin square design [21]. The test comprised two consumer evaluations: the first assessed consumer acceptability for 15 attributes on a 9-point structured hedonic scale (1= dislike extremely; 5 = neither like nor dislike; 9 = like extremely) and 3 attributes on a 9-point scale ranging from 1 (strongly disagree) to 9 (strongly agree). The consumer acceptability test targeted various attributes including overall_liking, appearance, inner color of scone, color, overall_taste, sweet taste, texture, savory taste, aroma/smell, aftertaste, flavor, softness, moistness, cohesiveness, outer crispiness, purchasing intention, recommendation, and familiarity.

The second assessment involved CATA questions where consumer panels selected terms that best described their tasting experiences. The drivers of liking for the CATA questions were derived from previous studies and a professional focus group [3,15,21,22,23,24,25,26,27]. The drivers of liking comprised 29 terms, including appearance, color, size, texture, harmonious pairing with jam, savory flavor, grainy flavor, unique flavor, oily, sweet, salty, familiar flavor, harmony of ingredients, convenient to eat, newness, traditional, unique texture, familiar, cohesiveness, outer crispy, inner color, nutty flavor, stuffy, crumbly, moist, healthy, thick throat feeling, residual sensation in the mouth, and others.

### 2.4. Statistical Analysis

A one-way analysis of variance (ANOVA) was utilized to compare consumer acceptability among the samples, establishing a significant difference threshold at *p* < 0.05. Following the detection of significant differences, Tukey’s multiple range test was applied as a post hoc analysis. A frequency analysis was performed to calculate the frequency of consumers’ answers to the CATA questionnaire. Chi-squared and Fisher’s exact tests were conducted to confirm the significance between samples. A correspondence analysis (CA) elucidated the relationship between the six samples and sensory terms evaluated by CATA, while partial least squares regression (PLSR) was employed to determine the association between the sensory terms assessed by CATA and consumer acceptability. All statistical analyses were performed using IBM SPSS (Statistical Package for Social Science, ver. 25.0, Chicago, IL, USA) and XLSTAT 2024.8 (Addinsoft, Paris, France).

## 3. Results and Discussion

### 3.1. Frequency of Sensory Characteristics and Correspondence Analysis of Six Scone Samples by CATA

**Figure 1:**
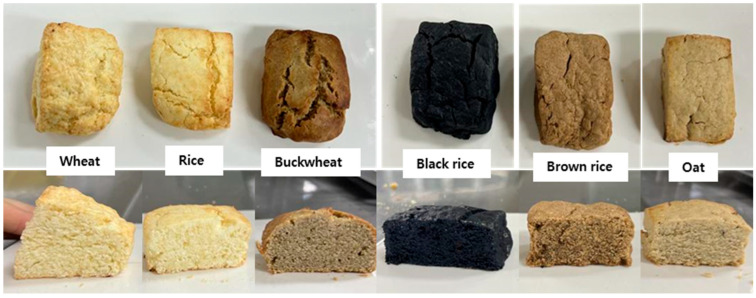
Scones with different flours (the scones should be about 4 cm wide, 5 cm long, and 2 cm thick).

Table 2 presents the frequency of 29 sensory terms for gluten-free scones based on CATA questions. Ten of those terms—appearance, color, texture, grainy flavor, sweetness, familiar flavor, newness, familiarity, and moistness—showed significant differences among samples (*p* < 0.001). Scones with wheat and rice flour were frequently chosen for their appearance, color, familiarity, and moistness compared to other varieties. The scone made with wheat flour exhibited the highest frequencies in texture, savory flavor, harmonious ingredient blend, familiarity, and moistness. This result indicates that consumers are more familiar with bakery products made from wheat flour. Additionally, the presence of gluten in wheat flour significantly influences the texture and quality of the bakery products from a sensory standpoint [28]. As illustrated in Figure 1, wheat and rice scones, similar in appearance and color to commercial scones, had higher frequencies in appearance, color, and familiarity attributes than others did. The black rice scone, distinguished by its color, appeared to have a high frequency of the ‘newness’ term. Furthermore, the black rice scone had the highest frequency of a ‘healthy’ attribute among the six scones, reflecting the perception that colored foods are positively correlated with health. However, the rest of the sensory characteristics had the lowest frequencies, indicating that the black color of the scone did not have a positive effect on the evaluation of the other organoleptic sensations.

**Figure 2:**
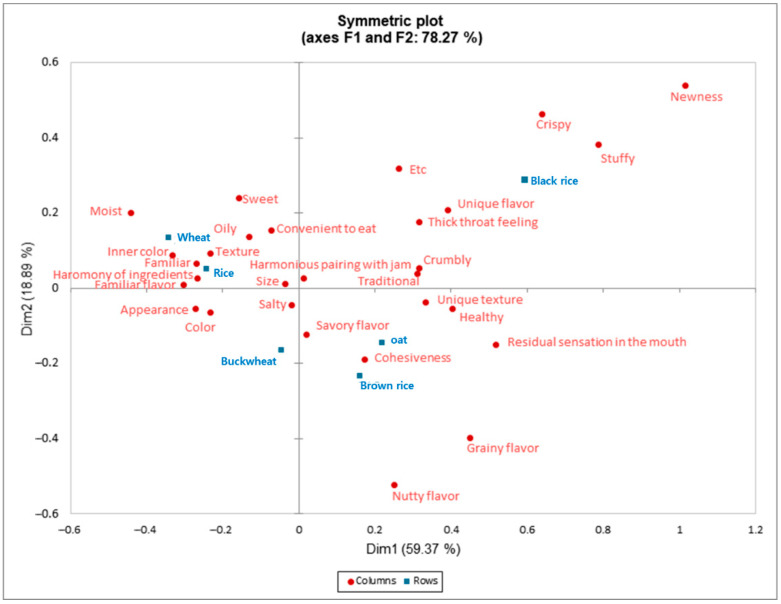
Correspondence analysis of six scone samples using 29 sensory attributes by CATA ^(1)^. Columns (•) mean attributes, rows (■) samples. ^(1)^ CATA, check-all-that-apply.

The correspondence analysis visually represented the sensory characteristics and sample distribution (Figure 2). The CA biplot explained 59.37% of the variance with Dim 1 and 18.89% with Dim 2, accounting for a total variance of 78.27%. Black rice, brown rice, and oat scone samples were positioned in the positive direction of Dim 1, influencing attributes such as crispiness, unique flavor, texture, cohesiveness, stuffiness, savory flavor, and grainy flavor. Conversely, wheat, rice, and buckwheat scone samples were located in the negative direction of Dim 1, affecting attributes such as moistness, oiliness, familiarity, texture, appearance, color, and harmonic ingredients. The attributes of texture, familiarity, appearance, color, and harmonic ingredients nearly coincided with wheat and rice scone samples. These scone samples exhibited high frequencies in appearance, color, and familiarity as noted in Table 2, indicating a close association with these attributes.

### 3.2. Consumer Acceptability

Table 3 presents the results of the consumer acceptability test for gluten-free scones. Consumer panels evaluated the attributes of overall liking, appearance, inner color of scones, color, overall taste, sweet taste, texture, savory taste, aroma/smell, aftertaste, flavor, softness, moistness, cohesiveness, outer crispiness, purchasing intention, recommendation, and familiarity using a 9-point hedonic scale. All attributes of the consumer acceptability test were significantly different among six samples (*p* < 0.001). Buckwheat and wheat scone samples had the highest consumer acceptability. The buckwheat scone sample was rated higher in acceptability of overall liking, inner color, overall taste, texture, savory flavor, sweet flavor, flavor, aroma/smell, softness, moistness, cohesiveness, outer crispiness, purchase intention, recommendation, and familiarity than that of wheat. The wheat scone sample showed the highest consumer acceptability for appearance, color, and aftertaste attributes. This result indicates that consumers are familiar with the appearance and color of scones made with wheat flour. In terms of appearance, the rice scone sample was judged to be the most similar to a regular wheat scone. Rice flour is the most widely replaced for wheat flour to make gluten-free products because it has little effect on the color and flavor of the product [28]. However, the results of our study showed that the attributes of the rice scone, except for appearance, were rated lower than those for buckwheat, black rice, and brown rice scones. The buckwheat scone sample attracted higher scores of consumer acceptability in terms of savory flavor, softness, and moistness than those of a regular wheat scone. Despite its unfavorable color, the black rice scone scored higher in consumer acceptability than the oat and brown rice scones. Healthy consumers choose gluten-free products based on emotional and psychological factors because they perceived black-colored foods as healthy [29]. Conversely, the oat scone sample had the lowest scores for consumer acceptability. The consumer acceptability of oat scone received lowest scores in color, savory taste, and textures such as softness, moistness, and outer crispness. Gluten-free bakery products usually tend to have dark colors and a hard texture because of a complex formulation [30]. The study of oat-based gluten-free cookies presents that there was a correlation between the chewing effort and low consumer acceptability [9]. The selection of bakery products is influenced by their appearance and texture in the mouth. As the demand for gluten-free products increases, gluten-free bakery products are being created from various gluten-free flours such as rice, sorghum, amaranth, corn, buckwheat, quinoa, and chickpea [12,18,31,32]. Buckwheat is one of the most commonly used ingredients in gluten-free bakery products in numerous studies because it is rich in nutrients including amino acids and polyphenols such as rutin [6,7,8,11,12,13,31,32,33].

Our findings align with previous studies by Dapčcević Hadnađev et al. [7], who examined the effect of substituting rice flour with buckwheat flour on the rheological properties and quality of cookies. They observed that cookies enriched with buckwheat flour were rated with higher overall acceptability than those made with rice flour. Additionally, they noted that cookies with buckwheat flour scored higher for pleasant smell and taste. Similarly, Sakač et al. [8] investigated the antioxidant capacity, mineral content, and sensory properties of gluten-free cookies made by substituting rice flour with buckwheat flour at levels of 10, 20, and 30%. Their findings indicated that cookies containing 20 and 30% buckwheat flour exhibited more favorable sensory properties (color and odor) compared to those with 0 and 10% buckwheat flour. Sedej et al. [6] also conducted a quality assessment of gluten-free crackers using buckwheat flour, comparing refined and wholegrain wheat and buckwheat crackers. They found that refined buckwheat crackers scored the highest in terms of appearance (shape, uniformity, surface), texture (structure, break, firmness), chewiness, and taste out of the four samples. Conversely, Joung et al. [13] studied the effect of wheat and six gluten-free flours (brown rice, buckwheat, corn, sorghum, teff, and black rice) on cookie quality, employing a consumer preference test to evaluate sensory properties including crust color, top grain, flavor, softness, sweetness, and overall preference. The buckwheat cookies received the lowest scores for overall preference and flavor, whereas black rice scones received the highest scores among the evaluations.

### 3.3. Relationships Between Sensory Attributes by CATA and Consumer Acceptability of Six Scones

**Figure 3:**
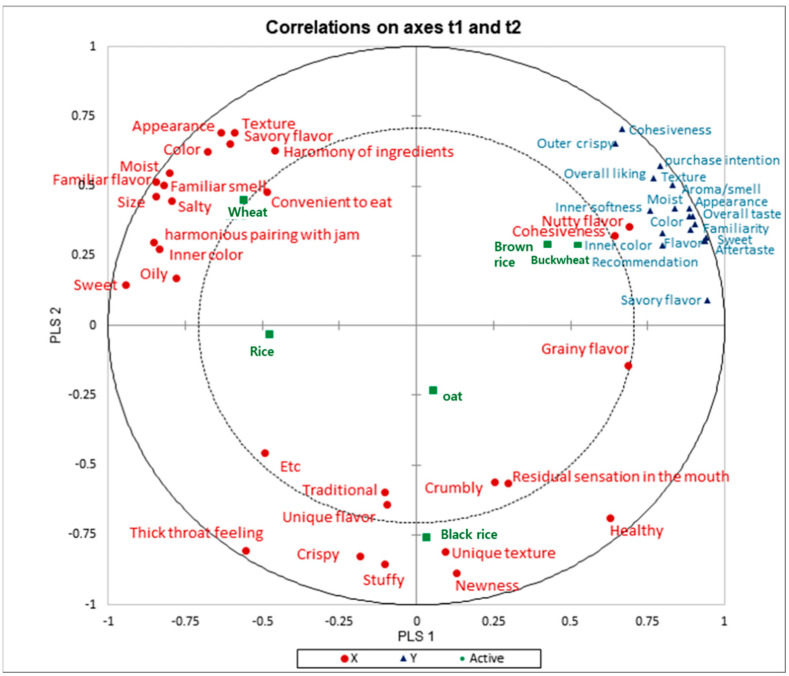
Results of PLSR ^(1)^ analysis between sensory attributes (•) by CATA and consumer acceptability (▲) of six scone samples (■). ^(1)^ PLSR, partial least squares regression analysis.

PLSR analysis was conducted to identify the relationship between sensory attributes by CATA and consumer acceptability of the samples. Figure 3 illustrates the relationship between sensory attributes by CATA and consumer acceptability of six scone samples. PLSR analysis is commonly utilized to clearly and visually depict relationships between two types of data [34,35,36]. We conducted an examination to derive the sensory characteristics of CATA that significantly influence consumer acceptability by applying the PLSR analysis. The results show a high positive (+) correlation between overall liking and purchase intention, with sensory attributes such as nutty flavor, cohesiveness, appearance, moistness, color, texture, and inner softness positively affecting acceptability. Attributes negatively affecting acceptability included thick throat feeling, unique flavor, and stuffiness. The black rice scone sample was closely associated with unique flavor, newness, crumbly texture, unique texture, and stuffiness. While scones are usually ivory or yellow in color, the black color of the scone due to the black rice was evaluated as a ‘newness’ factor, compared to a traditional scone. It is believed that these results correlate with the low consumer acceptability of the black rice scone sample. The oat scone sample was associated with a crumbly texture, residual sensation in the mouth, and grainy flavor, resulting in the lowest consumer acceptability. Conversely, the buckwheat and brown rice scone samples demonstrated high consumer acceptability due to positive attributes such as nutty flavor, cohesiveness, color, texture, overall liking, and overall taste. The study of buckwheat flour cookies from Dapčcević Hadnađev et al. [7] indicated sensory attributes contributing to higher consumer acceptability included the pleasant smell and taste of buckwheat cookies. In particular, Republic of Korean consumers prefer the ‘nutty flavor’ and perceive it as a positive sensory characteristic [34,35]. Sedej et al. [6] also demonstrated that consumer acceptability is influenced by the appearance and texture of gluten-free crackers made with buckwheat flour.

## 4. Conclusions

Gluten-free scones made with oat, black rice, brown rice, buckwheat, and rice flours were developed, and consumer acceptability was determined using CATA questions. Our results demonstrate the potential application of gluten-free flours in bakery products based on sensory properties. Buckwheat flour offers the highest potential for the production of gluten-free scones. Despite the growing need and demand for gluten-free products, there are limited gluten-free products that meet consumers’ expectation, and there is a lack of evaluation studies on appropriate and sufficient sensory characteristics and quality measures of gluten-free products. The results of this study are expected to provide fundamental data to aid in the development of better-tasting gluten-free products that meet consumer and market needs.

However, this study has some limitations. First, the gender imbalance in our study, which had more female than male participants, may affect how representative our findings are of the general population. In future studies, we will consider a more equal gender balance when recruiting participants. Second, we could not examine the physicochemical characteristics of gluten-free scones because our study was focused on substituting wheat flour with gluten-free flours and examining consumer responses using CATA question-based consumer testing. Substituting gluten-free flour for regular wheat flour in commercial products remains a challenge because gluten-free products generally scored lower for texture, taste, and consumer acceptability than those containing gluten. Furthermore, commercial gluten-free products tend to be more expensive. A significant challenge is developing technologies that can substitute affordable gluten-free products for gluten-containing ones while maintaining a similar texture and quality to meet consumer expectation and market demands. Continuous studies aimed at improving the quality of gluten-free products, such as adding hydrocolloids, protein isolates, gums, and modifying the flour, are ongoing. Additionally, it is essential to explore various gluten-free flours from plant materials with nutritional and functional ingredients and assess their consumer acceptability to satisfy consumer requirements.

Therefore, further research will be conducted to examine the nutritional quality, physicochemical characteristics, and sensory characteristics of gluten-free bakery products based on the result of this study.

## Figures and Tables

**Table 1:** Composition and mixing ration of scones.

Ingredient (Ratio)	Composition (g)					
	Wheat	Rice	Buckwheat	Black Rice	Brown Rice	Oat
Flour (47%)	150	150	150	150	150	150
Sugar (9.5%)	30	30	30	30	30	30
Salt (0.3%)	1	1	1	1	1	1
Baking powder (1.6%)	5	5	5	5	5	5
Butter (16%)	50	50	50	50	50	50
Whole egg (9.5%)	30	30	30	30	30	30
Milk (16%)	50	50	50	50	50	50

**Table 2:** Results for sensory attributes by CATA ^(1)^ of six scone samples (*n* = 100).

	Wheat (*n* = 100)	Rice (*n* = 100)	Buckwheat (*n* = 100)	Black Rice (*n* = 100)	Brown Rice (*n* = 100)	Oat (*n* = 100)	*p*-Value ^(2)^
	n	n	n	n	n	n	
Appearance	55	56	35	11	24	28	<0.001
Color	61	61	40	15	33	28	<0.001
Size	43	43	27	24	27	26	0.003
Texture	48	25	22	14	19	10	<0.001
Harmonious pairing with jam	41	41	18	25	31	26	0.001
Savory flavor	44	30	21	19	37	33	0.001
Grainy flavor	6	11	15	16	30	26	<0.001
Unique flavor	7	5	5	11	6	5	0.477
Oily	4	2	-	1	1	3	0.304
Sweet	18	20	4	9	7	7	<0.001
Salty	4	4	2	2	3	3	0.927
Familiar flavor	27	25	6	4	13	13	<0.001
Harmony of ingredients	15	7	7	3	5	5	0.019
Convenient to eat	24	7	9	10	10	6	0.001
Newness	2	4	3	22	6	6	<0.001
Traditional	4	8	4	8	7	5	0.670
Unique texture	3	7	5	7	5	7	0.777
Familiar	33	29	11	8	14	13	<0.001
Cohesiveness	4	4	9	5	6	2	0.317
Outer crispy	3	2	1	7	2	3	0.182
Inner color	9	13	3	2	3	5	0.005
Nutty flavor	5	5	9	4	15	11	0.030
Stuffy	3	2	-	9	3	6	0.017
Moist	32	20	9	5	7	6	<0.001
Crumbly	2	4	5	5	2	3	0.729
Healthy	18	17	25	33	26	28	0.074
Thick throat feeling	4	5	2	6	3	5	0.744
Residual sensation in the mouth	1	5	2	6	7	6	0.231
Others	2	1	1	2	-	2	0.771

^(1)^ CATA, check-all-that-apply; ^(2)^ *p*-value by Fisher’s exact test.

**Table 3:** Consumer acceptability of six scone samples using the 9-point hedonic scale.

	Wheat (*n* = 100)		Rice (*n* = 100)		Buckwheat (*n* = 100)		Black Rice (*n* = 100)		Brown Rice (*n* = 100)		Oat (*n* = 100)		*p*-Value ^(1)^
	Mean	SD	Mean	SD	Mean	SD	Mean	SD	Mean	SD	Mean	SD	
Overall liking	6.56 ^(2)a^	1.31	4.92 ^bc^	0.98	6.72 ^a^	1.68	5.16 ^bc^	1.03	5.17 ^b^	1.03	4.42 ^c^	0.74	<0.001
Appearance	7.77 ^a^	1.55	6.08 ^bc^	1.22	6.95 ^ab^	1.74	5.39 ^c^	1.08	5.80 ^bc^	1.16	4.09 ^d^	0.68	<0.001
Inner color	7.00 ^a^	1.40	5.83 ^b^	1.17	7.01 ^a^	1.75	5.54 ^b^	1.11	5.84 ^b^	1.17	4.14 ^c^	0.69	<0.001
Color	7.11 ^a^	1.42	5.89 ^b^	1.18	7.06 ^a^	1.41	5.67 ^b^	1.13	5.97 ^b^	1.19	3.98 ^c^	0.66	<0.001
Overall taste	6.40 ^a^	1.28	4.81 ^b^	0.96	6.78 ^a^	1.36	5.20 ^b^	1.04	4.90 ^b^	0.98	4.50 ^b^	0.75	<0.001
Texture	6.12 ^a^	1.22	4.98 ^b^	1.00	6.51 ^a^	1.30	5.08 ^b^	1.02	4.62 ^b^	0.92	4.59 ^b^	0.76	<0.001
Savory flavor	6.12 ^ab^	1.22	4.94 ^c^	0.99	6.47 ^a^	1.08	5.95 ^ab^	1.19	5.49 ^bc^	1.10	4.88 ^c^	0.81	<0.001
Sweet taste	6.07 ^a^	1.21	4.72 ^bc^	0.94	6.20 ^a^	1.03	5.39 ^b^	1.08	4.94 ^bc^	0.99	4.34 ^c^	0.72	<0.001
Aftertaste	6.16 ^a^	1.23	4.45 ^c^	0.89	6.10 ^a^	1.02	5.27 ^b^	1.05	4.69 ^bc^	0.94	4.15 ^c^	0.69	<0.001
Flavor	6.08 ^a^	1.22	4.55 ^bc^	0.91	6.36 ^a^	1.06	5.27 ^b^	1.05	4.89 ^bc^	0.98	4.46 ^c^	0.74	<0.001
Aroma/smell	6.21 ^a^	1.24	5.00 ^b^	1.00	6.49 ^a^	1.08	5.52 ^b^	1.10	4.82 ^b^	0.96	4.95 ^b^	0.82	<0.001
Softness	5.98 ^b^	1.20	4.57 ^c^	0.91	6.63 ^a^	1.11	4.77 ^c^	0.95	4.73 ^c^	0.95	4.51 ^c^	0.75	<0.001
Moist	5.82 ^b^	1.94	4.78 ^c^	0.96	6.53 ^a^	1.09	4.68 ^c^	0.94	4.55 ^c^	0.91	4.35 ^c^	0.73	<0.001
Cohesiveness	5.65 ^ab^	1.88	5.09 ^bc^	1.02	5.92 ^a^	0.99	4.88 ^c^	0.98	4.61 ^c^	0.92	4.75 ^c^	0.79	<0.001
Outer crispy	5.13 ^ab^	1.71	4.76 ^bc^	0.95	5.73 ^a^	0.96	4.56 ^bc^	0.91	4.24 ^c^	0.85	4.21 ^c^	0.70	<0.001
Purchase intention	6.04 ^(3)a^	2.01	4.32 ^bc^	0.86	6.58 ^a^	1.10	4.79 ^b^	0.96	4.42 ^bc^	0.88	3.66 ^c^	0.61	<0.001
Recommendation	5.93 ^(3)a^	1.98	4.08 ^bc^	0.82	6.34 ^a^	1.06	4.60 ^b^	0.92	4.21 ^bc^	0.84	3.46 ^c^	0.58	<0.001
Familiarity	5.90 ^(3)a^	1.97	4.24 ^b^	0.85	6.50 ^a^	1.08	4.67 ^b^	0.93	4.47 ^b^	0.89	3.33 ^c^	0.56	<0.001

^(1)^ *p*-value by one-way Anova. ^(2)^ 1 = Dislike extremely; 5 = Neither like nor dislike; 9 = Like extremely. ^(3)^ 1 = Disagree strongly; 5 = Neither agree nor disagree; 9 = Agree strongly. ^a–d^ Indicates significant differences between the samples for each attribute by Tukey’s multiple range comparison at α = 0.05.

## Data Availability

The data presented in this article are available from the corresponding authors upon request.
